# Health and legal literacy for migrants: twinned strands woven in the cloth of social justice and the human right to health care

**DOI:** 10.1186/s12914-017-0117-3

**Published:** 2017-04-13

**Authors:** Bilkis Vissandjée, Wendy E. Short, Karine Bates

**Affiliations:** 1 Faculty of Nursing, Université de Montréal, PO Box 6128 Station Centre-Ville, Montréal, QC H3C 3J7 Canada; 2 Université de Montréal, Faculty of Nursing, Université de Montréal, PO Box 6128 Station Centre-Ville, Montréal, QC H3C 3J7 Canada; 3 University of Queensland, Faculty of Humanities and Social Sciences, St. Lucia, Queensland 4072 Australia; 4 Department of Anthropology, Université de Montréal, PO Box 6128 Station Centre-Ville, Montréal, QC H3C 3J7 Canada

**Keywords:** Justice, Ethics, Equity, Human Rights, Gender, Health literacy, Legal literacy, Empowerment, Migration

## Abstract

**Background:**

Based on an analysis of published literature, this paper provides an over-view of the challenges associated with delivering on the right to access quality health care for international migrants to industrialized countries, and asks which group of professionals is best equipped to provide services that increase health and legal literacy. Both rights and challenges are approached from a social justice perspective with the aim of identifying opportunities to promote greater health equity. That is, to go beyond the legal dictates enshrined in principles of *equality*, and target as an ethical imperative a situation where all migrants receive the particular assistance they need to overcome the barriers that inhibit their *equitable* access to health care. This assistance is especially important for migrant groups that are further disadvantaged by differing cultural constructions of gender. Viewing the topic from this perspective makes evident a gap in both research literature and policy. The review has found that while health literacy is debated and enshrined as a policy objective, and consideration is given to improving legal literacy as a means of challenging social injustice in developing nations, however, no discussion has been identified that considers assisting migrants to gain legal literacy as a step toward achieving not only health literacy and improved health outcomes, but critical participation as members of their adoptive society.

**Conclusion:**

Increasing migrant health literacy, amalgamated with legal literacy, aids migrants to better access their human right to appropriate care, which in turn demonstrably assists in increasing social engagement, citizenship and productivity. However what is not evident in the literature, is which bureaucratic or societal group holds responsibility for assisting migrants to develop critical citizenship literacy skills. This paper proposes that a debate is required to determine both who is best placed to provide services that increase health and legal literacy, and how they should be resourced, trained and equipped.

## Background

For universal health care to be truly realised in a community, everyone in that community must be informed of their legal right to health care, and equipped to access, understand and critically participate in it. Despite having diverse places of origin and resettlement trajectories, in host nations where universal medical care is provided on the premise of equality and social justice for all, migrants share with locally-born citizens the right to access health services [1]. The designation “migrant” is used here to signify any person born in a country other than their adoptive place of permanent, or temporary residence, and encompasses immigrants, humanitarian migrants, and temporary residents [2]. Considering the particular context of migrant health, this paper begins by considering the ethics and intent of universal access to health care, and the ethic of fairness which engenders an obligation to address those social determinants that constitute barriers to universal health care [3, 4].

While universal health care systems recognise patient and migrant diversity, and aim to respond equitably to the range of circumstances experienced by all groups of women, men, girls and boys [5–7], it is the position of these authors that any host country medical system will be, to at least some extent, “foreign” for all migrants. The level of assistance an individual migrant or migrant population requires in order to equitably access health care, will be variously influenced for example by the size and resources of their migrant community or family cohort, their category of entry and migration status, and integration trajectories [2, 8–11]. Some migrant groups for whom the host medical culture is closely aligned with their pre-migration experience, require only minimal guidance in order to gain the requisite health system literacy to access and engage in medical care. However, for other migrant cohorts whose previous life experiences incorporate vastly different cultural mores and modes of health care practice, acquiring even a basic level of understanding of their right to health care is so challenging as to constitute a barrier to health. Unravelling the complex and dynamic factors that assist or hinder particular migrants as they seek to access the health services they need in their daily lives, therefore requires an understanding not only of the systems of the adoptive country, but also of the complexities associated with migrants’ diverse life trajectories. Of particular import are the diverse challenges and needs experienced by differently gendered groups of migrant women and men. A recognised social determinant of health, gender exacerbates the barriers to care encountered by those migrants whose gender identity, beliefs and prior life experiences differ from those of the host culture and health system norms regarding sex and gender practices [12–14].

In this multifaceted context, advancing migrant health literacy to assist in overcoming barriers to accessing care goes far beyond developing language comprehension and an individual’s ability to schedule an appointment. For health and legal literacy to support a migrant community’s critical engagement with the barriers to care and citizenship rights that they encounter, requires reciprocal education efforts, with migrants learning to employ the host medico-legal system, and the system being adapted to respond sensitively to migrant’s cultural conceptualisations of health [15–17]. It is this critical and reflexive literacy that ultimately allows migrants to take control of their experienced social determinants of health to improve both their social participation and quality of life [11, 17, 19].

Despite the complex interrelation between migrant’s understanding their legal rights to health care, and developing their ability to work with the medical system to achieve equitable levels of care, the published literature appears yet to explore the challenges associated with: a) development of legal literacy for migrants including a migrant’s awareness of their right to health care under the law; b) the interrelatedness of legal and health literacy within the differential trajectories of women and men migrants; c) migrants’ ability to critically use those rights to empower their lives; and d) defining a scope, delivery mechanism and imprimatur for a professional group to assist migrants to increase their legal and health literacy, and therefore self-advocacy skills. The aim of this paper is therefore to prompt discussion in these four areas.

## Ethics grants a *right* to health care

Discussion of the desirability and challenges of delivering universal health care is predicated on a belief, enshrined in international human rights agreements, that it is morally fair that all individuals have “rights to health and to a standard of living adequate for health” [4] (p. S150), [6, 19]. This is a belief, inspired by social justice and based on an ethical position with respect to the human right to “equality of opportunity to enjoy the highest attainable level of health” [4] (p. S150). In realising these ideals, the domain of human rights becomes multifaceted, as the practical out-workings of an individual’s right to health care are negotiated within the contexts of national public health agendas, spheres of economic trade rights, and particularly the individual’s right to privacy, freedom of religion and self-determination [6, 20–22]. Despite this multiplicity of expression, the premise in the field of public health is that health care should be available to all [4, 7]. Furthermore, governments that are signatories to international agreements such as “The Universal Declaration of Human Rights”, “The International Covenant on Civil and Political Rights” and “The International Covenant on Economic, Social, and Cultural Rights” [21] have moral and legal obligations to reduce social injustices, including mitigating barriers to accessing medical care, particularly for those most vulnerable groups within their population [19, 20]. As a result, the moral imperative to deliver universal health care is often also legislated within a jurisdiction, and so becomes a domestic legal obligation, which translates into a legal right for the individual to be able to access care [1]. Inhibiting a population or group’s access to health care by processes of discrimination or injustice, be they overt or inadvertent, is not only unlawful, but creates for that disadvantaged group a sense of exclusion, social illegitimacy and separation from the moral community [20, 21]. So, while provision of health care occurs within a network of cooperative processes involving bureaucracy, professionals of multiple disciplines, and economics, actions in the health sphere impact migrant identity in ways that can have ramifications beyond generating health inequities [1, 23].

Where some groups in a society experience *avoidable* systematic differences in health outcomes resulting from unjust social determinants of health, this unfair imbalance is termed “health *in*equity” [3]. The aim of a just society is “health equity” and the seminal definition is offered by Whitehead [24]: “Equity in health implies that ideally everyone should have a fair opportunity to attain their full health potential and, more pragmatically, that none should be disadvantaged from achieving this potential, if it can be avoided.” (p. 433). Implicit in this statement of “fairness” is a valuing of the desirability of social justice for all. It should be noted that this discussion focuses on the human right to “health care” and not a more esoteric “right to health”. As argued by Eleftheriadis [21], the health of an individual or group is determined by a diversity of factors, biological and social determinants of health, not all of which are related to the availability of health care. The definition of “health care” used here follows Rumbold [6] in combining *positive* care—provision of intervention and response to medical needs, and *negative* care or care that is protective against risks to health, such as providing information regarding dietary risks, and legislative prohibitions which serve to limit exposure to passive smoking or failure to disclose HIV serology before unprotected sexual intercourse for example. In either case universal health care is made available in forms that are equitable and respond to need [6].

In the United States, researchers have noted that the terminology “health disparity” was initially used to highlight the lower health outcomes observed in disadvantaged racial and ethnic groups, and that use of the term simply to describe all difference in health outcomes between different racial groups shifts the primary focus away from addressing social disadvantage [20]. Whether focussed on socio-economic constraints or racial inequities, the legal principle of providing health care according to need not privilege, elicits a dialogue within a social justice paradigm. According to the report of the Commission on the Social Determinants of Health [3, 5], such social inequalities can be corrected only if a particular society makes the required efforts to activate changes at the structural level. And so, while delivering health equity is the target, the particularities of any debate are made only in reference to the specific, contextualised ramifications within a particular setting [25].

A large body of literature focussing on health outcomes for migrant communities, emphasises patterns of “acculturation” (for example: [26–31]). However, the term “acculturation”, taken to mean “the extent to which individuals embrace “mainstream” versus ethnic culture”, particularly with respect to adopting local dietary norms, is considered by others to be problematic in seeking to understand the health outcomes of migrant communities [13] (p. 974.) Viruell-Fuentes et al. [18] noted in the United States for example that “factors such as immigration policies, labour practices, neighborhood characteristics, and racialisation processes intersect… (re)producing poverty, and racial discrimination… all of which likely influence the health of immigrants above and beyond the influence of… cultural traits” (p. 2100). In Canada, Hankivsky [32] pointed out that migrant health is “affected by dislocation, isolation, loss of identity, culture and meaningful employment” (p. 1714). Beyond ignoring the complexity of the migrant’s experience, words such as “acculturation” have the power to “other” and objectify those most needing care [33]. By discounting the very significant social and structural factors which impact migrant health, the “acculturation” approach places the onus, in some cases moral blame, onto the individual and community, constructing a state of cultural vulnerability [18]. This paper therefore rejects the acculturation approach, and instead considers that health care provision for migrants should be understood via a social justice lens which recognises the barriers created by intersections of migration trajectory, gender hierarchies, race, prejudice, class, and education.

From this discussion, it can be seen that the conditions that foster ill-health are no longer solely within the purview of the health care professional: “These problems require solutions at the interstices of social, political, cultural, and economic domains where public health’s role shifts from acting alone to engaging as a coordinator and motivator of various, sometimes unusual, partners in sectors not directly responsible for health” [19] (p. 655). Where social determinants of health are considered to be due to social inequality and injustice, the role of health care professionals is to advocate for social change that addresses the root causes of social inequality [3]. It is therefore insufficient that health care be made available in a manner that addresses the normative needs of the wider community. Instead, Willen [23] argues that the social justice factors that impact health outcomes must be addressed simultaneously from the perspectives of 1) justice in equitable rights and entitlements, 2) moral deservedness and need, and 3) empirically demonstrated (im)practicalities of access (p. 809). Taken together these dynamic influences constitute Social Determinants of Health and when combined with environmental, biological and lifestyle or behavioural factors, begin to encompass the variables that constrain health aspirations [34]. According to the World Health Organisation, social determinants of health “are the circumstances in which people are born, grow up, live, work and age, and the systems put in place to deal with illness. These circumstances are in turn shaped by a wider set of forces: economics, social policies, and politics” ([5], see also [35]). Therefore, to different degrees in particular contexts, social determinants of health are understood to include “income and social status; social support networks; education; employment/working conditions; social environments; physical environments; personal health practices and coping skills; healthy child development; gender; and culture” [36]. It can therefore be seen that while access to health care is itself a determinant of health, other determinants both directly impact on health and also limit an individual’s ability to access health care, resulting in a compounding effect and creating successive, inter-exacerbating layers of vulnerability [37]. It is therefore important to recognise the complexities involved in attempting to unravel causal connections, or the different mechanisms that may operate to cause or moderate disadvantage [36].

While selected authors have sought to develop models outlining the intrinsic interdependence between determinants of health, others have attempted to single out the unique contribution of each. In particular, researchers have focused on the differential life trajectories of women and men as they are framed and inculcated by structural dynamics [12, 14, 32, 38–40]. Gender equity for example is a concept that allows for an understanding that sometimes different groups of women and men have different health needs, resulting from the intersection of a variety of social and biological factors [41]. That is, an individual’s ability to *access* health care is determined at any moment by an intersection of factors originating within the medico-legal system and within the migrant’s life course: some factors facilitate access, some impede, all impact on the migrant’s health at that time and as a consequence, later in life. This dynamic, mutually constitutive relationship between social factors, which occurs and impacts as a result of socially instituted power relations, is described by feminist scholars as “intersectionality” [14, 42, 43]. In the context of considering access to health care, intersectionality allows for recognition of power dynamics which drive social inequalities, and the interrelationships between experienced social realities such as gender, sexual orientation, race, language, age, physical/mental ability, economics, education, life trajectory, knowledge and experience [20]. One of the outcomes of this wide body of research is a diversity of literature probing the contributing differential and intersecting life circumstances which result in some enjoying better health than others. In this regard, social position has been identified as a key factor in an individual becoming aware that they have a right to quality health care, and then accessing that care. The knowledge gained from considering these multiple perspectives is essential to understanding intersectionality and thereby improving provision of care in a context made yet more complex by diverse cultural understandings of sex, gender and social justice [44–46]. Adopting an overview of social determinants of health allows the researcher and policy-maker to observe shifts in power structures and gain the perspective required to identify and intervene in the processes which create, maintain and reproduce health inequities at the level of the individual and migrant community [47].

## Justice grants *access* to health care

Legally, an equal entitlement to health care is available to all who qualify as deserving within the definitions of that jurisdiction [23]. However, as law is a socio-political construct built on categories derived from and within social life, premises of law shift in response to social change, and rights manifest differently between national contexts [48, 49]. As a result, the specific health care related legal rights and particular responsibilities constituted in the adoptive nation are, at least initially, outside the experience of the migrant [7]. Furthermore, the interrelationship between law and state as instruments of a specific culture empowered to define the application of justice, imparts the law with the breadth of responsibility, power and imprimatur which can elevate its views above consideration of the particular social circumstances in which sex, gender and migration are intersecting determinants of need [7, 23, 50]. Rocher [51] stated that: “to understand the level of effectiveness of a law involves tracing the diversity of its effects and impacts, intended or unintended, planned or accidental, direct or indirect, foreseen or unexpected, social, political, economic, or cultural” (p. 133). The importance given here to the addressing the legal obstacles to be overcome when accessing medical care rests on the premise that it is only when women and men, newcomers or not, are able to decode the language of law and of health, that the principle of universal access to health and to justice can truly be said to have been applied [15, 52, 53]. In other words, “[t] he intervention has to be accessible, acceptable, effective in, and used by the most disadvantaged group within that population to be truly effective at reducing inequities in health” [54].

Some individuals and population groups, routinely navigate the processes of their health care systems as a matter of course, while for others this presents a significant, multifaceted challenge. As stated, for a migrant the challenges can begin with endeavouring to gain a knowledge of the details of their legal rights and responsibilities with regards to health care: information that is not always available in a form or language that they can understand [55, 56]. In a period of uncertainty and widening gaps between social extremes, health care and justice systems need to reinforce their respective capacities, and those of the women and men who use their services, by focusing on a more effective system for accessing information: a necessary condition for realizing the right to ready access [11, 55–57]. Schuler [50], and Schuler and Kadirgamar-Rajasingham [58] defined legal literacy in the context of developing self and social empowerment for women in lower middle income countries, and it is argued that the same concept applies here: legal literacy is “[t] he process of acquiring critical awareness about rights and the law, the ability to assert rights, and the capacity to mobilise for change” [50] (p. 5). So at the most basic level, for a migrant to access their right to health care, acquiring legal literacy means that they first need to acquire some awareness of their legal rights, of how to appropriate those rights and to work to overcome any encountered barriers to care. To go further and actively express agency in engaging and participating in their health care, to understand what is happening to them, to confront issues, and to exercise self-determination about their health care, requires the migrant, or migrant community, to have attained a level of literacy well beyond a basic awareness of their legislated rights. An example of this might be that beyond their right to access care, patients should be aware that they have legal rights during care, as a minimum typically including rights to provide informed consent, to a second opinion, and to privacy [56]. Establishing a trust relationship is important to achieving sensitive and healing clinical medical encounters, however, having developed trust in the medical sphere, the migrant further relies on socio-legal information gained from health care professionals who are not necessarily trained in terms of the social justice needs of their patients [59]. Yet statements of patient rights provided by medical practices are often written by legal advisors, and are presented using legal terminology that is often unapproachable for any native-born lay individual, let alone the migrant [56]. The role of the internet in health communication is beyond the scope of this current paper, however it should be noted that some government health services now provide significant levels of information on the internet regarding patient rights and responsibilities. This is an area requiring further study with regard to utilisation by migrants and the variability, appropriateness and accessibility of information in specific jurisdictions. For example, the www.settlement.org webpage provided by Immigration, Citizenship and Refugees Canada for new arrivals in the province of Ontario is available in English and French, with numerous documents available in 40 languages [59]. This webpage offers migrants separate sections on health services, their rights and responsibilities with regard to accessing health care, and their legal rights, all in plain language. However, a level of language capability, and access to and familiarity with the internet is still required [57].

Whether newly arrived or long established, economic or humanitarian categories, for a migrant to acquire sufficient legal literacy to apprehend their rights in particular health care contexts is akin to learning an additional new vocabulary and medico-legal culture [11]. This is an additional learning and adaptive process that is often beyond the resources of women and men going through a diversity of integration experiences and facing medical encounters in their adoptive society [11]. Furthermore, the power and bureaucratic formality of the law can create an additional risk as migrants who are the living human subjects of their legal rights may be reduced by the “system” to the static and inhuman position of being considered “objective facts” or impersonal objects of care [7, 33, 55]. Such an objectification completely overlooks the gendered identities and socio-political life courses of real women and men seeking redress, and taken to extreme has similarities to the objectification of refugees described in Malkki‘s seminal work “*Speechless Emissaries: Refugees, Humanitarianism and Dehistoricization*” [60]. It has been documented that administrative hierarchy, which is ostensibly responsible for ensuring a clear process of proper procedures for claimants, may on occasion prove counterproductive as bureaucratic processes require a level of literacy that proves detrimental to a claimant [18]. It is furthermore noted that information provided in a form that is unintelligible to the audience is technically not available [56]. When accessing care and bureaucratic due process, any individual is required to provide documentation to substantiate their right to care, for the migrant this adds the potential for incurring: direct costs associated with the need to produce verified and current official documents, possibly incurring legal fees; indirect costs such as lost work time, transport and childcare; and the stress brought on by the complexity of dealing with the para-legal system. These factors can combine to progressively further erode the migrant’s ability to access their legal right to health care. Perhaps remarkably, despite this, Conley and O’Barr [62] reported cases where women and men going through periods and processes of frustration with justice are still able to make their objective case, developing and presenting local citizenship skills, and drawing on inner personal strength and in some cases community reserves of social support.

Another intersection of legal rights and health care needs which presents particular challenges for migrants is raised by language comprehension and the need for interpreters [63, 64]. Even speakers of a language as a mother tongue can struggle with: regional terminology and colloquial usage; literacy and numeracy; following signage within a health complex; understanding appointment details; completing administrative documents and insurance forms; and understanding written materials regarding their diagnosis, medication and self-care [55, 56]. Bischoff [65] identifies that where the patient and physician do not share a language, patients are less likely to be given follow up appointments, are less likely to attend follow up appointments, and are less likely to complete prescribed treatment regimens (p. 541). Rudd [56] reports that individuals with low literacy, who have lower levels of understanding of their condition and its management, are more likely to be hospitalised (p. 73). Migrants may have a legal right to the services of an interpreter, however this imposes a cost, requires prior scheduling, and even if an interpreter is available and the physician is trained in working in this way, there can be struggles to explain a medical condition and history, with medical terminology, and with issues of power and status [55, 56, 65]. This is just one further example where even if migrants critically apprehend their legal and health rights, unique dynamic confluences of legal, health and social determinants such as ethical independence of interpreters, migration trajectory, employment status and gender, can and do impact both on a migrant’s health status, and their ability to access health care [1, 23].

### Gender and the right to equitable access to health care

The role of sex and gender can be pivotal with regards to female, transgender, intersex, lesbian, gay or bisexual migrants, first in the individual’s choice to seek medical care, in understanding their rights to be free of prejudice and discrimination, and feeling able to communicate equitably and honestly with care personnel who are trained to understand their particular situation and needs [56]. For these particularly socially vulnerable groups, access to health care is not equitable, and as a result diseases may not be diagnosed early, infections can spread and overall community health outcomes differ [23, 34, 38]. To deliver justice, the administration of health care organizations needs to address gender implications of actions to ensure the needs of all gender groups receive equitable, needs based redress [3, 38, 66].

While “sex” refers to *aspects of the body* with a biological or reproductive definition, “gendered social embodiment” and sexual orientation are *co-determined* by biology and culture, and “gender ideologies” which structure “gender identity” and social relations are *derived culturally* and are in constant flux [32, 67]. Within any society gender ideology is contingent, arising in history, and today is interwoven with the fabric and dynamics of world culture [67]. As notions of gender, gendered social embodiment, hierarchies and identities vary with shifting cultural contexts, they differentially affect access to and use of resources, often building from the perceived differential capabilities and needs of different groups of women and men [32, 66]. These definitional distinctions are important when seeking to address universal health care. For example, in order to be universal it is vital to avoid conflating “gender” with “women”, as was done by the Commission on Social Determinants of Health, thereby ignoring gender specific constraints impacting men and other gender groups such as the Lesbian, Gay, Bisexual, Transsexual and Intersex communities [3, 66, 68]. Sexual and gender identities are complex constructs, highly dependent on social context [38], and as such, inequalities in health care settings that are caused or exacerbated by differing cultural understandings of gender identity based factors are also socially produced and therefore avoidable and addressable. And yet sexism and gender minority discrimination remain culturally embedded, and although often legally prohibited are sometimes subtly manifest [3, 4, 69]. For migrants, the complexity is compounded as they seek to translate differing cultural gender ideologies, expressions and expectations, and to understand their legal right to freedom from gender based discrimination. VanderPlaat and Teles [70] went as far as to assert that assuming a collective, gender-blind experience, and opting not to gender disaggregate data, is itself a form of discrimination which directly impacts the health rights of diverse gender groups (p. 36).

So, while all the intersecting social determinants of health described above are contextual and compound in different ways for the individual and their community, it is the position of these authors that sex and gender today remain the overarching canvas upon which other social factors come to bare in a gender-nuanced manner: with Shields “[w] e do not suggest that gender is always and everywhere the most important social identity, but it is the most pervasive, visible and codified” [14] (p. 307). We are in no way arguing for gender-specific medicine that defines bodies into binary differences based on sex [71], nor that gender is always the major site of discrimination in an individual’s life [32]. Rather we posit that in this neoliberal, patriarchal time in human history, social bureaucratic contexts have a gender lens that means that men, women, transsexual and intersex individuals have different experiences of the constellations of power relations that constitute their medical journeys. It is clearly important, as argued by Hankivsky [32] that care professionals consider both the possibility that gender is impacting health outcomes, and are alert to situations where apparent gender impacts are masking other underlying social factors (p. 1717). The institutions of health care are gendered, for patients, carers and administrators and “[a] nalysis needs to consider simultaneously the shape of the gender order, and its historical transformations, the pattern of institutional and interpersonal relations, and the body-reflexive practices in which health consequences are produced” [67] (p. 1679). As Celik et al. [12] stated: “Gender sensitivity means that health professionals are competent to perceive existing gender differences and to incorporate these into their decisions and actions” (p. 143). And the human and justice implications are significant: Hammarström et al. [41] noted for example the overwhelming and consistent evidence linking gender inequality with patterns of mental illness, morbidity and mortality (p. 185).

The arguments highlighted in this paper are therefore anchored in a perspective that the disparities between women and men, as well as between groups of women and men, which often stem from trends embedded within society, are reflected in their daily lives and health experiences. We would argue with Hammarström et al. [41] that a legal right to “gender equality” describes only a situation of no discrimination between women and men, while the objective of universal healthcare should be to develop a “gender equity” focus which delivers women’s and men’s health needs, whether similar or different, and irrespective of gender (p. 185). For the migrant, this consideration will need to encompass at least two gendered cultural milieus and vulnerable interstices of transition and change. As alluded to above, identity and vulnerability are at constant crossroads where a variety of spaces and resources connect. A specific context is created in which “social relations of inclusion and exclusion [are] constantly evolving, with social relations being built and reconstituted in territories and spaces in which everyone does not end up a ‘subject of law’ on an equitable basis” [47, 55].

## On legal literacy, health literacy and empowerment

To this point this discussion has referred generically to a migrant’s legal and health literacy as being of vital import to their ability to overcome social determinants of health and the limitations of their ignorance of “the system” in accessing health care in their new country. Health literacy, similarly to legal literacy, consists of a certain ability to understand, assess, access and communicate selected information in order to engage with complex demands in complex contexts for specific purposes in reference to one’s well-being [11, 15, 52, 61, 72–74]. In his seminal paper, Nutbeam [16] recognised three levels of health literacy, where greater health literacy results in an individual not only being able to access health care, but to understand, and ultimately to critically engage in ensuring just outcomes. These levels were defined by Nutbeam [16] as: functional health literacy, where the individual can communicate sufficiently to understand health risks and is able to use the health system; interactive health literacy, where the individual is able to act on the health information received; and critical health literacy, where the individual has the confidence and skills to critically engage the health care system and exert agency over the care obtained, confronting social injustice either for themselves or their community (p. 265). It is at the level of critical health literacy that the individual is an active participant in their health care and has the skills and confidence such that social determinants of health particular to that individual’s community can be challenged. For this to occur, the migrant must be a critical participant in the dynamic discursive process not just a passive recipient of knowledge [56]. We would argue, that from a justice perspective, the objective is not only critical health literacy but also critical legal literacy, where the individual is sufficiently informed both of their legal rights and the needs they have in their health situation, and is empowered to accept control for those rights being addressed and for the actions taken with regards their health. VanderPlaat and Teles [70] stated that: “the collaboration between health and human rights begins here, with the struggle to decide whose view of health will control [it]” (p. 35). It is at this point Nutbeam [16] pointed out, once critical health literacy is achieved, that health care becomes something that is done with people, not to them (p. 265).

South American educator, Paolo Freire’s work on education and the raising of consciousness served as the basis of an intervention model that still influences health professionals who seek to empower individuals and communities through building critical health literacy [15, 52, 73–75]. Freire’s work emphasised that people educate themselves together, in their shared relationship with reality [75]. By establishing a connection between situations of domination, language, values, cultural alienation and political alienation, the objective targeted by Freire’s approach was the achievement of a critical consciousness in order to be able to analyse reality and its contradictions in order to become emancipated from it [16]. As well, for Freire [75], problematic situations had to be defined by the persons directly affected by them if the desired goal is a transformation of the relationship with the world and a redistribution of power.

Nutbeam [16], Freire [75], Estacio & Comings [15] and Zanchetta et al. [74] are clear in identifying that the ideal is for all disadvantaged people to attain critical health literacy where they are able to make a decision fully aware of its legal and medical implications. VanderPlaat and Teles [70] noted the centrality of an individual’s participation in decision making about their health and the health of their community, and the connection of that participation to realisation of their human rights (p. 35). That it is from a position of critical health literacy that unfair and unjust social determinants of health can be identified, challenged and changed [15, 16]. But it could be questioned whether, in a health system with limited resources, the medico-legal system has a responsibility to invest the time and resources in educating every vulnerable migrant to attain critical health literacy. To question from a political perspective whether it is absolutely necessary that every migrant achieve the level of knowledge of system literacy required to challenge injustice and advocate for changes to social determinants of health. Certainly the literature is robust and evidenced that critical health literacy is vital in order to achieve empowerment, to understand a community's notion of what constitutes health for them in their symbolic and relational paradigm, and for an individual or community to tackle the political task of changing social determinants of health (including for example: [11, 15, 16, 18, 40, 52, 72–74, 76]). But the question can still be read in from economic [77] and philosophical standpoints [21]: is critical health literacy a necessary ability for everyone?

From a purely economic standpoint, Smith, Mitten and Curshaw [77] argued that the priority for the Canadian government to address social determinants of health is so significant, that if necessary funding from other medical services should be reallocated to redressing social disadvantage as a means of reducing long term strain on the health care system. This argument aligns with the findings of Vázquez et al. [7] which identified that a failure to invest in health literacy leads to increasing costs as a result of demands on emergency medical services and loss of productivity (p. 244).

According to Nutbeam [16] the benefit of critical health literacy for the individual is “increased resilience to social and economic adversity” (p. 266). And yet it could perhaps be argued, that disadvantage people needing medical care may be more than satisfied by attaining interactive health literacy. At the interactive level, the system is sufficiently understood to enable access to needed care, to consent to and trust the care received. This argument is investigated by Jordan, Buchbinder and Osborne [72] who attended emergency departments in order to interview patients about their opinion of the health literacy skills that would have assisted them in the case of their medical event (p. 36). The user responses aligned well with interactive health literacy criteria, although the desire for increased personal assertiveness suggests a critical health literacy goal [72]. The level of patient desire to assert control over the process of their medical care will be community and individual specific, and certainly the designation “migrant” does not imply an homogeneous experience or aspiration. Migrant needs and literacy aspirations are certainly expected to vary with context, social support, adoptive country and country of origin, particular disadvantage and so forth. But the responses obtained by Jordan, Buchbinder and Osborne [72] appear to indicate that not every user of a health care system is going to want the skills to address community wide social injustices.

In response however, we might consider a hypothetical case of a migrant woman with endometriosis who suffers regular severe pain with menstruation. As a women’s medical disorder, discussion of her condition may be taboo in her country of origin or adoptive country. Interactive health literacy would enable her to access care and medication, to understand her disease, and to consent to treatment procedures. However, she may perhaps be discriminated against at work for her recurring illness, a condition that she hesitates to explain as it is a private women’s matter. Such a woman could benefit from a level of critical health and legal literacy that empowers her to access any sick leave entitlements, and to seek legal redress if her employment is terminated. She may need the confidence to speak openly with medical professionals or to seek an alternate opinion if her experience is denigrated as being normal for a woman. The ability to access her right to appropriate quality care and challenge unfair social structures suggest that in this case, the woman would benefit from critical health and legal literacy to defend her own individual position. And if she were later to start a support group, a safe place for migrant women with other gynaecological issues, then that would constitute a community critical health literacy intervention, challenging gendered injustice by providing social support.

In a concept analysis of critical health literacy, Sykes [73] noted that since Nutbeam’s writing [16], much of the discussion of critical health literacy has ignored the community social justice component, focusing on the individual and their participation in the health care they receive. While Sykes [73] advocated a return to recognising the social justice component of critical health literacy at the level of the community, these authors would further argue that in our hypothetical case above for example, the woman’s knowledge of her employment rights with regard to illness, to her right to seek another opinion and be heard, constitute not only critical health and legal literacy, but a defence of her individual human and democratic citizenship rights as a tenet of social justice. This hypothetical situation would need to be tested but its potential, and the economic arguments of Smith, Mitten and Curshaw [77] and Vázquez et al. [7], at least challenge any argument against providing critical health literacy to all migrants.

### Gaining health and legal literacy and empowerment

In order to be able to ‘adopt’ rules and regulations associated with the uptake and application of one’s rights to health and legal systems as full-fledged citizens, women and men as members of their adoptive society need to both inform themselves and be informed [11, 57, 78, 79]. The issue however remains as to the allocation of the task of educating vulnerable new members of society in achieving legal and health literacy. The literature surveyed did not contemplate the question as to the most appropriate professionals for assisting migrants in this task. Where contemplated the presumption was that nurses would assist migrants in understanding their rights [40, 61, 76]. But the question remains as to the reasonableness of that presumption, and the roles and responsibilities that health care and legal system workers carry in terms of making an active contribution to increasing health and legal literacy in a gender and culture sensitive manner among migrants, and for that matter for every citizen seeking their respective services.

If health care workers, specifically nurses are to take a key role in migrant’s acquiring health literacy and becoming culturally adept, it is necessary to consider the clinical best practice changes that would be required to strengthen both the level of health and legal literacy for migrants and the pedagogical skills of nurses. An uptake of such complex responsibilities requires sharp and sensitive communication skills from health care and legal system workers so as to be sure that users do in fact gain a clearer understanding. Nurses would need appropriate training and time allocation within their care responsibilities [40, 80, 81]. It is not easy to establish effective dialogue in fast paced clinical or legal settings, and it would be essential that the all parties are clear in regards to the ethics of respective roles, responsibilities and expectations in regards to rights of women and men, especially when levels of literacy are limited [61, 76]. Even so, of necessity and need nurses do take on the role of health literacy education within a medical response or care event [61, 76]. The level of attention and effort to assist in developing literacy by nurses described by Messias Hilfinger, McDowell, & Dawson Estrada [61], is beyond what might be expected to be possible given time pressures which typically constrain nurses to assisting with only functional health literacy advice [40]. The time poor professionals simply may not have the time to spend with individual patients, engaging in dialogue, understanding the patient’s context, world view and understanding of health so as to educate, inform and increase their literacy to the critical level necessary for and self-determination.

It could be argued that the task of health education is best undertaken by trained and resourced educators whose key role is educating people to interactive health literacy. Curricula would need to be tailored to the needs and worldview of each disadvantaged group every time, having been developed with members of the community to address their cultural conceptions of health and wellbeing, cognizant of their material, symbolic and relational context [15]. And a specific, significant budget for training and program development would be required, in line with the recommendations of Smith, Mitten and Curshaw [77] and the experiences reported by Vázquez et al. [7].

This would not however obviate or negate the important role of nurses and other health care professionals in raising awareness of their rights, and health literacy for their patients [61]. Porr, Drummond and Richter [76] described the work of nurses helping young single mothers to understand their legal and medical rights. These are vulnerable women who may never choose to attend a formal session run by an educator and could be at risk of not receiving any assistance with legal and health literacy if nurses were to stop providing that support. This highlights the need for collaboration between nurses and program organisers to tailor delivery to the specific needs, context, priorities and vulnerabilities of that disadvantaged group, to be innovative, to be aware of the situation of their lives, their needs and conceptions, symbols and sources of meaning [15]. Location and timing of sessions would need to respond to the priorities and commitments of the community: “how can you expect us to fill our brains when our stomachs are empty?” [15] (p. 1064). The work of Estacio and Comings [15] on increasing the health literacy of indigenous people highlighted the importance of health and legal literacy training being responsive to the recipient group’s understanding of health and incorporating their insights into program development in ways that are “based on the community’s conceptualisation of health [and law] in their material, symbolic [includes language] and relational environment” (p. 1059–1060).

As first identified by Freire [75], and emphasised in the work of Estacio and Comings [15] and Zanchetta et al. [74], in all these considerations, in order to deliver the social justice objective of tackling social determinants of health it is important that members of the disadvantaged group are educated to critical health literacy, so that they are aware of the social determinants of health that are limiting them, and so they can decide to be advocates and negotiate change. Pivotally, this includes Freire’s notion of critical consciousness of oppression, where “health literacy is perceived as an educational tool to be used not only to empower but also to inform and enlighten individuals and communities” [52] (p. 4). Having members of the community engaged in developing literacy curricula will enhance that opportunity. An option to be considered in future research may be an alliance between health care professionals, community development and social workers, for social participation and dialogue with affected groups. To assist people and their communities to gain the legal and health literacy necessary to express their own “voice” and exercise agency in addressing the unfair and inequitable social determinants of health important to them.

## Conclusion

### Weaving the cloth of literate health equity

The question of migrant health and legal literacy is anchored in the complexity of individual and community enablement in action. One of its premises is to develop sustainable strategies to allow for increased literacy through sensitive health education which encourages a more informed participation in decision-making in terms of one’s health situation and legal position [11, 15, 16]. The approach of using critical literacy as a route to realising the democratic rights of citizenship develops for the migrant local cultural negotiation skills, and generates positive self-esteem as migrant men and women combine their own personal strengths with local social resources. But it also holds the risks of making the ‘subjects’ take on the burden of responsibility for obtaining the skills and resources to address their condition, whereas, particularly in the case of new arrivals or small migrant groups, they are often powerless when facing social forces determining their health and legal situations. Literacy, rising from the idea of a dialectical educational process [15, 52, 75], is then a double-edged approach. Even though it carries a great emancipating potential with gender and migration experience sensitivity, it nonetheless could, through a simple reprioritisation of funding, reinforce social injustice and vulnerability by deflecting the onus and responsibility back onto the migrant and their community.

The struggle against social inequalities is clearly a matter of justice; but it is primarily a matter of will and economics, a question of promoting a positive demonstration of and engagement with migrant citizenship. Access to health care and justice as a right can be improved by establishing an environment in which the ‘subjects’ of rights, the care recipients and the care givers, engage in a dialogue free of gender-based biases and where the acknowledgment of rights is accompanied by concrete, effective and equitable measures: where migrants can become mediators in negotiating their own health outcomes.

Sustained effort is required to develop models for decision making in complex contexts and to translate them into everyday practices of shared decision making involving women, men, clinicians and legal workers addressing gender, culture, migration and linguistic challenges.

“*But as long as we are ignorant of the natural man [sic], it is in vain for us to attempt to determine either the law originally prescribed to him [sic], or that which is best adapted to his [sic] constitution. All we can know with any certainty respecting this law is that, if it is to be a law, not only the wills of those it obliges must be sensible of their submission to it; but also, to be natural, it must come directly from the voice of nature*’ [82], … there must be justice.
